# Translating Promoting Factors and Behavior Change Principles Into a Blended and Technology-Supported Intervention to Stimulate Physical Activity in Children With Asthma (Foxfit): Design Study

**DOI:** 10.2196/34121

**Published:** 2022-07-25

**Authors:** Annette Brons, Katja Braam, Aline Broekema, Annieck Timmerman, Karel Millenaar, Raoul Engelbert, Ben Kröse, Bart Visser

**Affiliations:** 1 Digital Life Center, Amsterdam University of Applied Sciences Amsterdam Netherlands; 2 Department of Information and Computing Sciences, Utrecht University Utrecht Netherlands; 3 Centre of Expertise Urban Vitality, University of Applied Sciences Amsterdam Amsterdam Netherlands; 4 Faculty of Health, Sports and Social Work, Inholland University of Applied Sciences Haarlem Netherlands; 5 Topsport Amsterdam Amsterdam Netherlands; 6 Play and Civid Media, University of Applied Sciences Amsterdam Amsterdam Netherlands; 7 Department of Rehabilitation, Amsterdam University Medical Center, University of Amsterdam Amsterdam Netherlands; 8 Department of Computer Science, University of Amsterdam Amsterdam Netherlands

**Keywords:** intervention mapping, technology-supported intervention, mobile health, mHealth, tailoring, exercise, cocreation, social participation, gamification, mobile app, web-based dashboard, chronic disease, mobile phone

## Abstract

**Background:**

Children with asthma can decrease the impact of their disease by improving their physical activity (PA). However, health care providers lack interventions for children with asthma that effectively increase their PA levels and achieve behavior change. A technology-supported approach can positively influence PA and physical functioning in children.

**Objective:**

The aims of this study were to develop a technology-supported intervention that facilitates health care providers in promoting PA for children (aged 8 to 12 years) with asthma and to systematically describe this developmental process.

**Methods:**

Intervention mapping (IM) was applied to develop a blended and technology-supported intervention in cocreation with children with asthma, their parents, and health care providers. In accordance with the IM framework, the following steps were performed: conduct a needs assessment; define the intervention outcome, performance objectives, and change objectives; select theory-based intervention methods and strategies; create components of the intervention and conduct pilot tests; create an implementation plan; and create an evaluation plan.

**Results:**

We developed the blended intervention *Foxfit* that consists of an app with a PA monitor for children (aged 8 to 12 years) with asthma and a web-based dashboard for their health care provider. The intervention focuses on PA in everyday life to improve social participation. *Foxfit* contains components based on behavior change principles and gamification, including goal setting, rewards, action planning, monitoring, shaping knowledge, a gamified story, personal coaching and feedback, and a tailored approach. An evaluation plan was created to assess the intervention’s usability and feasibility for both children and health care providers.

**Conclusions:**

The IM framework was very useful for systematically developing a technology-supported intervention and for describing the translational process from scientific evidence, the needs and wishes of future users, and behavior change principles into this intervention. This has led to the technology-supported intervention *Foxfit* that facilitates health care providers in promoting PA in children with asthma. The structured description of the development process and functional components shows the way behavior change techniques are incorporated in the intervention.

**Trial Registration:**

International Clinical Trial Registry Platform NTR6658; https://tinyurl.com/3rxejksf

## Introduction

### Background

Physical activity (PA) is important for children with a chronic disease [1,2]. Asthma is the most frequently diagnosed medical condition in children [3]. For children with asthma, PA can positively affect their asthma control by improving their physical fitness. This can reduce the threshold of triggers causing asthma symptoms, which in turn may lead to decreased use of medication and increased quality of life [1,4-6]. Besides the positive effects of PA regarding physical fitness, stimulation of PA in children with asthma is also important for their psychosocial functioning, because PA is often intertwined with social life. Children with physical disabilities are less involved in social activities compared to their healthy peers, and children with asthma often feel left out of the group [7-9]. Despite all benefits of PA, children with asthma—especially girls—seem to be less physically active than their healthy peers [10-13].

In the Netherlands, children are treated for asthma according to the *Dutch Pediatric Society* guidelines for asthma in children [14]. This treatment includes taking medication, receiving information, and having regular check-ups. Children with asthma who are inactive are referred to health care providers, such as pulmonary nurse practitioners and pediatric physical therapists. They provide health education, encourage children to increase their PA levels, and pay attention to social participation and children’s self-confidence regarding PA. Moreover, they educate them on how to cope with exercise barriers and provide exercise training. Such training interventions, which include, for instance, swimming and aerobic exercises, are shown to be effective in increasing cardiovascular fitness and in improving pulmonary function of children with asthma [6,15-19]. However, health care providers indicate that they lack interventions that result in maintenance of improved PA levels. This is because existing training programs do not aim to achieve behavior change in children with asthma [15,17,20].

This raises the question as to how PA levels can be both increased and maintained in children with asthma who are inactive. Various studies and reviews show that effectiveness of PA stimulating programs can be increased by technology-supported interventions [21-29]. Although most studies focus on adults, several studies show that a technology-supported approach can have positive effects on PA and physical functioning in children and adolescents as well [30,31].

Features of technology-supported interventions, especially behavior change interventions, are often inadequately described [23,24]. Therefore, it is difficult to compare interventions and their effects. To ease and improve this comparison, it is important to describe the design process and the developed components of an intervention [32,33]. Several frameworks are already used to describe interventions that stimulate PA, such as the MRC (Medical Research Council) framework to stimulate PA in older adults, the IDEAS (Integrate, Design, Assess, and Share) framework to promote PA in adults, the CeHReS (Centre for eHealth Research) framework to promote PA in people with depression, and the intervention mapping (IM) model to stimulate PA in patients with heart failure [33-40]. For our intervention, we chose the IM model, because it explicitly differentiates between personal and environmental factors affecting PA, and it takes into account creating an implementation and evaluation plan. Moreover, IM was already successfully applied for developing health interventions for children [41-43]. The framework enables systematic development of evidence-based interventions and enhances adoption, implementation, and maintenance of the developed intervention [39].

### Objectives

As part of a project that aims to promote PA in children (aged 8 to 12 years) with asthma, we attempted to develop an intervention that supports health care providers in stimulating PA in children with asthma using the IM model. In a preliminary study, we explored promoting factors for PA in children with asthma according to children with asthma themselves, their parents, and their health care providers [44]. In this study, we applied the IM model to develop a technology-supported intervention that incorporates both these promoting factors and behavior change principles that were discovered. We aimed to transparently report the development process and the final components of the intervention using IM to meet the demand of sufficiently describing technology-supported interventions [32,33]. We answered the following research question: “How can scientific evidence on stimulation of PA, the needs and wishes of future users, and behavior change principles be translated into features of an intervention that facilitates health care providers in promoting PA in children with asthma?”

By systematically describing the translation from requirements to functional components and our specific design and implementation choices, we give insight into the background of our intervention and ease comparisons with other interventions. In addition, we report the contribution of future users, because the intervention was developed in cocreation with children with asthma, their parents, and health care providers.

## Methods

### IM Model

The IM protocol includes six steps: (1) conduct a needs assessment in which a distinction is made between personal and environmental determinants; (2) define the intervention outcome, performance objectives, and change objectives; (3) select theory-based intervention methods and strategies; (4) create components of the intervention and conduct pilot tests; (5) create an implementation plan; and (6) create an evaluation plan.

### Step 1: Needs Assessment

To assess the health problem, background information and scientific evidence regarding children with asthma, the impact of low levels of PA, and available interventions had to be gathered according to the IM model. In our preliminary study, 3 stakeholder groups were questioned to explore promoting factors of PA in children with asthma. To do so, concept-mapping sessions were held with 25 children with asthma (aged 8 to 12 years), 17 parents of children with asthma, and 21 health care providers of children with asthma. The health care providers were lung nurse practitioners and pediatric physiotherapists, experienced in behavior change and supporting PA in children with asthma. The resulting factors were labeled as either personal or environmental factors according to the Physical Activity for People with a Disability model [45]. More details regarding the needs assessment can be found in our preliminary study [44]. On the basis of this needs assessment, we defined the aim of the intervention. The reported factors will be used in subsequent steps of the IM framework, such as designing the program’s requirements and functional components.

### Step 2: Intervention Outcome, Performance Objectives, and Change Objectives

To examine the target behavior, we created a logic model of change according to the IM framework. First, we defined the program outcomes based on the stakeholders’ reported clusters in the needs assessment. They describe the desired changes in the behavior and the environmental conditions that are required to reach the goal of the intervention [39]. Second, we translated the program outcomes in performance objects based on the stakeholders’ reported supporting ideas in the needs assessment. Those indicate the required behavior to reach the goal of the intervention. On the basis of these performance objectives, change objectives were defined which indicate the necessary behavior to accomplish the required behavior change and environmental conditions.

### Step 3: Selecting Theory-Based Intervention Methods and Strategies

On the basis of the needs assessment and the logic model of change thus created, we selected several methods and strategies that were translated into design considerations for the intervention. This was performed in a stakeholder session with a pediatric pulmonologist, a pediatric physiotherapist, and researchers specialized in exercise behavior in children, behavioral change, health technology, and game development. The final selection was a product of mutual agreement.

### Step 4: Creating and Pilot-testing the Intervention’s Components

On the basis of the needs assessment, logic model of change, and the design considerations, we formulated several requirements for the intervention to be developed. With a multidisciplinary team involving health care providers, data scientists, game designers, and software developers, we translated the requirements into functional components. This translation was performed in 2 sessions and the final list of requirements was a product of mutual agreement. To develop those components, we performed an agile method consisting of several design and develop iterations. Each iteration lasted for 2 weeks, and there were 10 iterations in total.

In the first 3 iterations, we created story lines, mock-ups, and user journeys resulting in a prototype. In a stakeholder session, this product was tested by children with asthma and their health care providers. Children who participated in the needs assessment were asked whether they wanted to participate in this stakeholder session as well. Children who were diagnosed with asthma and aged between 8 and 12 years could be included. The 3 participating children were aged, on average, 9.3 (SD 1.5) years, and 2 of them were female. One of the children was diagnosed with severe asthma, one of them had mild asthma symptoms, and one of them had moderate asthma symptoms. The 4 participating health care providers were all pediatric physiotherapists with >5 years of work experience with children with asthma. In the stakeholder session, the participants individually tested the prototype and shared their experiences with the researchers. The children were asked to focus on the app’s functionalities, the story line, and the usability of the PA monitor, whereas the health care providers were asked to focus on the dashboard’s functionalities and the clarity of the graphs showing the children’s PA. Afterward, the researchers compared the experiences of the participants and translated them into requirements for improvement of the prototype. In the remaining 7 iterations, these improvements were made, and the prototype was further developed into a usable product.

### Step 5: Creating an Implementation Plan

In a stakeholder session with a pediatric physiotherapist, a pediatric pulmonologist, and a lung nurse practitioner, we examined the health care provider’s daily practice and the current health care situation for children with asthma who are inactive receiving care regarding PA. We explored the available time and skills of the health care providers, to support children with asthma who might benefit from PA with an intervention.

### Step 6: Creating an Evaluation Plan

For a blended intervention, it is important to evaluate the usability and feasibility in children’s everyday life and in their health care provider’s daily practice. Therefore, we selected methods to evaluate user experiences, adherence, and usability of both children and health care providers. In addition, to evaluate the possible impact of the intervention on children, we selected methods to look into the self-perception of PA among children, their social participation, and their enjoyment of PA.

### Ethics Approval

The Amsterdam University Medical Center Medical Ethical Committee approved the research protocol with trial number METC 2017 191. The study was registered in the International Clinical Trial Registry Platform with trial number NTR6658 on August 21, 2017.

## Results

### Step 1: Needs Assessment

Several studies show that children with asthma benefit from PA. Increased levels of PA are related to both improved cardiovascular fitness and quality of life [4-6,16,19]. However, children with asthma experience limitations in PA and social activities because of their disease [7-10,46]. Reported explanations for these experienced limitations are, for instance, inaccurate symptom perception of airflow limitation, misinterpreting healthy exercise-induced shortness of breath as an asthma attack, and low self-efficacy in relation to PA [47,48]. Parents endorse the limitations that children experience and report parental fear for exercise-induced asthma, challenges with asthma management, and lack of trust in teachers or sport coaches as parental barriers [49,50]. Increasing self-esteem and improving knowledge about PA in relation to asthma and medication are reported as strategies to reduce children’s perceived limitations in PA [51,52].

**Figure 1:**
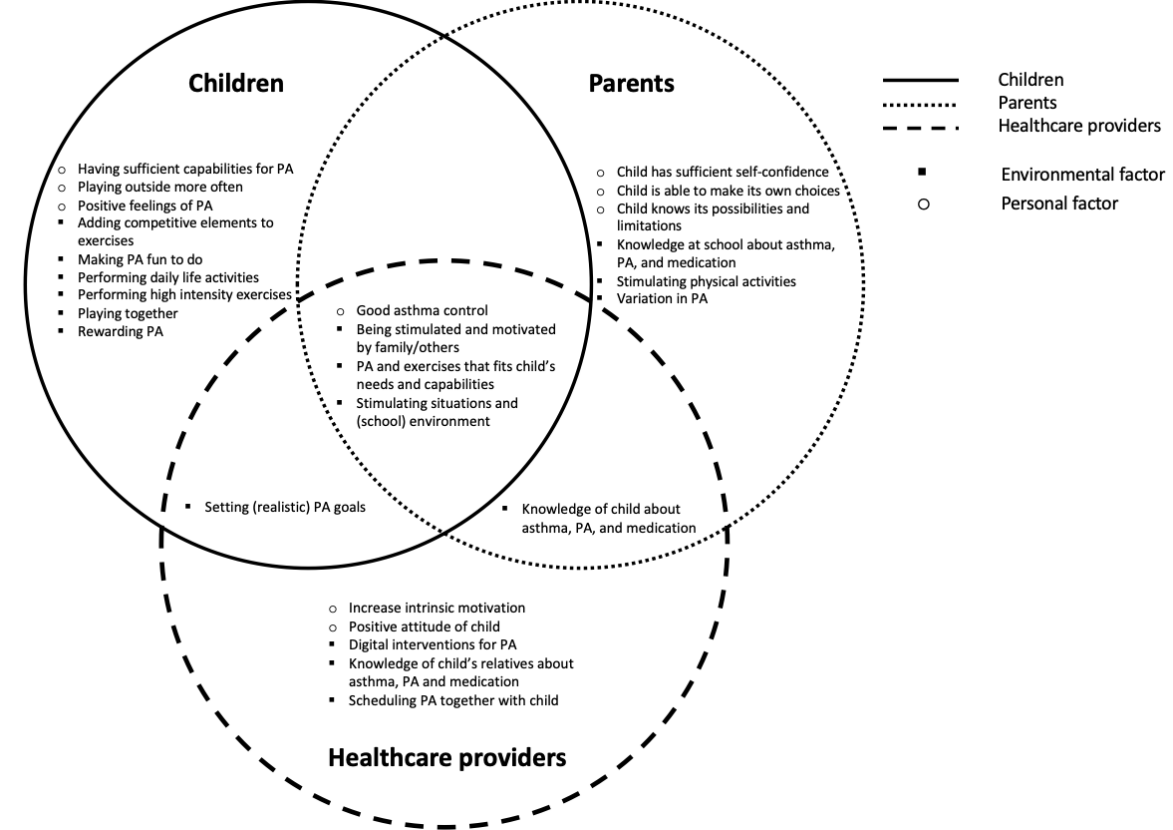
Venn diagram in which the reported factors of all stakeholders are combined. The circles indicate the stakeholder groups. The factors are listed in random order (reprinted from Brons et al [44] with permission from the author). PA: physical activity.

Figure 1 shows differences and similarities among all reported factors of the 3 stakeholder groups. Factors that were rated as very important were getting positive feelings of PA, playing together, being stimulated and motivated by relatives, good asthma control, making PA enjoyable, rewarding PA, tailored PA, playing outside more often, setting realistic goals, relatives of child have sufficient knowledge about asthma in relation to PA, child itself has sufficient knowledge about asthma in relation to PA, digital interventions for PA, increasing extrinsic motivation, positive attitude of child, scheduling PA together with child, and variation in PA.

The aim of the intervention was defined as follows: the intervention needs to support health care providers in improving PA levels in children with asthma who might benefit from increased levels of PA. The intervention will be developed for children who are inactive, with mild to moderate asthma symptoms, who are referred to a health care provider, either a lung nurse practitioner or a pediatric physiotherapist, to improve their PA levels.

### Step 2: Intervention Outcome, Performance Objectives, and Change Objectives

All program outcomes, along with their corresponding performance and change objectives, are shown in Table 1.

**Table 1:** Logic model of change: program outcomes with their corresponding performance objectives and change objectives^a^.

Program outcomes and performance objectives			Change objectives
**Increase PA^b^ participation**			
	Use active transport instead of motorized transport	Walk or cycle more often	
	Participate in sports activities at school	Communicate about willing to join sports activities Inform school about the disease to create a safe environment	
	Participate in social activities with PA elements	Register for social PAs, even when having some doubts Find a trusted buddy that can help when needed	
	Participate in sports activities	Register for sport activity that fits capabilities Inform the coach about the disease to create a safe environment	
	Resist sedentary behavior	Search for enjoyable alternatives for sedentary time Screen time should be reduced (by parents)^c^	
**Improve knowledge about asthma and medication in relation to PA**			
	Gain knowledge about impact of PA and medication on asthma control	Information about asthma and medication in relation to PA should be provided by health care provider or parents^c^	
	Gain knowledge about PA possibilities despite having asthma	Information about PA possibilities when having asthma should be provided by health care provider^c^	
**Increase asthma control**			
	Good medication adherence	Take medication as prescribed	
**Increase self-esteem regarding PA**			
	Experience positive PA feelings	Learn individual capabilities and limitations	
**Increase perceived joy during PA**			
	Play together	Ask friends or family to participate in PA together Schedule PA in family agenda	
	Include competition element	Add competitive elements to PA PAs with competitive elements should be available^c^	
	Introduce variation in PA	A variety of PAs should be accessible^c^	
	Earn rewards	Rewards should be given for performed PA^c^	
**Provide a stimulating environment**			
	Receive support from parents	PA should be actively supported by parents^c^	
	Create a supportive school environment	PA should be actively supported by school^c^ Access to PAs should be given by school^c^	
**Provide tailored PA**			
	Provide an adaptive tool	A flexible tool that adapts to the child’s specific needs and wishes should be available^c^	
	Receive tailored feedback	Individual feedback based on monitored PA behavior should be given by health care provider^c^	
**Learn to set realistic goals**			
	Set PA goals	Think about own PA behavior and formulate a goal to improve the current behavior Obtain insights into one’s own PA behavior Coaching regarding setting realistic goals should be provided by health care provider or parents^c^.	

^a^Change objectives regarding personal factors (shown in all cells unless otherwise indicated by footnote c).

^b^PA: physical activity.

^c^Change objectives regarding environmental conditions.

### Step 3: Selecting Theory-Based Intervention Methods and Strategies

The following three design considerations were formulated: (1) support behavior change, (2) blended technology, and (3) integration in everyday life.

To start with, the intervention should support behavior change regarding PA. Gamification is frequently used in the improvement of health and well-being and has shown to be particularly effective in behavior change regarding PA [53]. Gamification is defined as “the use of game design elements in non-game contexts” [54]. Conceptually, gamification combines design elements for behavior change techniques from persuasive technologies, intrinsically motivating qualities from serious games, and methods for tracking individual behavior from personal informatics [53,55-59]. However, in practice, most gamified eHealth applications merely implement short-term engagement through extrinsic rewards [60]. For children with a chronic disease specifically, the nonsignificant results of several serious games aiming to increase PA might be explained by the lack of incorporating behavior change theories in the game [61]. To reach the full potential of gamification, it is necessary to design tools based on theories that support the overall goal, behavior change, and the effects of game mechanics [60].

To maintain improved levels of PA, children must develop new habits. An important factor in habit formation is rewarding the required behavior [62]. Rewarding increases positive feelings in relation to the behavior, in our case performing PA [63]. To develop intrinsic motivation, such positive feelings are important [64]. Increasing self-efficacy is also shown to be effective, for instance, through action planning and shaping knowledge [65-67]. Moreover, goal setting, monitoring and feedback are shown to be effective persuasive methods for improving PA behavior [67-69]. Personal coaching from health care providers, for instance, with the widely used motivational interviewing technique, might enhance the behavior change process [70,71].

Second, the intervention should use blended technology, meaning that individual use of digital technology is combined with face-to-face interaction with a health care provider. Digital technology was shown to be valuable for improving health in children and supporting behavior change [30,72]. However, the results of such digital technologies can be optimized by offering them in combination with personal coaching [30]. Personal coaching is important to provide feedback and support goal setting, which are both identified as success factors when behavior change is aimed [67-69]. Moreover, personal coaching by health care providers reinforces a tailored approach, which was also identified as a success factor of eHealth interventions that induce health behavior change [73-76]. Both a tailored approach and support of a health care provider are important supporting factors of shaping knowledge, which is a frequently used technique in supporting healthy behaviors [67]. Children with asthma specifically need to learn more about the positive relation between PA and asthma control, how to properly take asthma medication, and the difference between healthy shortness of breath and an asthma attack [47]. Moreover, feedback and knowledge regarding accurate symptom perception is important for perceived exercise limitations. Some children report substantial discomfort when there is limited bronchoconstriction, whereas others report no symptoms even if severe obstruction is present [48].

Finally, it is important that use of the intervention and performing PA could be integrated in everyday life of the users. Although participation in everyday activities is a vital part of children’s development, children with physical disabilities or chronic diseases are less involved in such social activities than their healthy peers [8,58,77,78]. Children with asthma report that they often feel left out of the group [9]. Integration in everyday life is therefore important from 2 perspectives. On the one hand, we have to prevent that using the intervention creates a special situation as this might reinforce children’s feeling of being different and excluded. On the other hand, PA is often intertwined with social life. By focusing on PA in everyday life situations instead of sports specifically, children might experience better social participation.

### Step 4: Creating and Pilot-testing the Intervention’s Components

#### Requirements of the Intervention

The requirements of the intervention are as follows:

1. Facilitate behavior change regarding PA by supporting goal setting, action planning, behavior execution, self-monitoring, and evaluation.
2. Monitor children’s PA so that both children and their parents can obtain insights into their PA behavior.
3. Connect the technology used by the children with the technology used by the health care providers. That way, health care providers can obtain insights into children’s PA behavior and can provide feedback. However, participating children should not see PA behavior of other children to prevent them from comparing themselves with each other. Children’s PA possibilities vary by the severity of their asthma symptoms, and their motivation might be lowered when they see other children achieving PA goals that are not achievable for themselves.
4. Promote PA in everyday life to increase social participation instead of focusing on sports activities.
5. Include learning elements to improve children’s knowledge about asthma in relation to PA. This should be presented in an attractive and stimulating way, because children with asthma themselves did not report improving their knowledge as an important stimulating factor.
6. Reward both exhibited PA and the effort undertaken to extrinsically motivate children. Although intrinsic motivation is preferred over extrinsic motivation to achieve behavior change, extrinsically motivated PA can yield positive feelings such as experiencing fun and feeling competent. Those positive experiences are in itself important factors to become intrinsically motivated.
7. Include adaptive components for a tailored approach such as personalized goal setting, planning, and education. Personalized goals are important to adapt to children’s specific situation.
8. Minimize required screen time for the intervention. There should only be functional screen time, because screen time induces sedentary behavior and therefore defeats the purpose of the intervention.

#### Functional Components of the Intervention

##### Structure

The final intervention, named *Foxfit*, consists of 3 technical elements that meet all the requirements: (1) a PA tracker that monitors children’s performed PA time and intensity, (2) the *Foxfit* app on the child’s smartphone, and (3) the *Foxfit* web-based dashboard for health care providers. Because the intervention is blended, children receive personal coaching and feedback from their health care provider every week during a coaching moment. In the gamified story, the children are a fox that wants to travel from the moon to a planet. The child can do so by gaining points by performing PA.

In the following description of functional components, it is explicitly stated when components were added or changed because of feedback on the tested prototypes. To emphasize the evidence-based components, Table 2 shows the mapping from methods regarding the design consideration, such as behavior change principles, to the final functional components.

**Table 2:** Translation from methods in the 3 design considerations to functional components of the intervention.

Design consideration and method		Functional component of the intervention
**Support behavior change**		
	Monitoring	Activity monitor that shows PA^a^ Points in the app that represent PA levels and bonus activities Overview of performed activities over the last week
	Gamified story	PA behavior, goals, and rewards are translated to an attractive story
	Rewards	Points for PA behavior based on activity tracker Bonus points for being aware of PA behavior Trophies from the health care provider for effort and positive attitude
	Action planning	Personal daily and weekly schedule with PAs
	Shaping knowledge	Tips and information as attractive drawings with supporting text Tips and information from the health care provider during the weekly meetings
	Goal setting	Personal daily and weekly goals in the form of PA points Personal PA goal for the entire duration of the intervention
**Blended technology**		
	Personal coaching and feedback	Weekly meeting with health care provider
	Tailored approach	Goals, activity schedules, suggestions for PA, and tips and information are tailored
**Integration in everyday life**		
	Include all PAs	Suggestions for PAs Activity tracker monitors both general PA and specific sports activities
	No special situation	Usable without having the app with you (synchronize afterward)

^a^PA: physical activity.

##### Goal Setting

A total of 4 PA goals can be listed up on the dashboard and the most important one can be marked as the main goal, such as being able to play as a hockey field player instead of goal keeper for half a match. Moreover, a weekly goal can be set. Children can see both their main and weekly goals on the home screen of the app anytime.

##### Rewards

On the basis of the measurements of the activity tracker, children receive points for their performed PA. Children also receive rewards for their effort, because children that tested a pilot version indicated that they did not only want to be rewarded for actual performed and measured PA but also for a positive attitude and for trying without sufficient results. Therefore, their shown effort is rewarded by their health care providers with either a bronze, silver, or golden trophy.

##### Self-monitoring

The activity tracker facilitates self-monitoring of PA. The app shows the received points visualized as the location of the fox on its way to the planet. In addition to self-monitoring, the child’s PA behavior is visualized in graphs on the health care provider’s dashboard. As requested by health care providers who tested the prototype, these graphs are easy to interpret, because they do not have much time to prepare. Moreover, at the request of health care providers, the graphs also show the relation between PA behavior and the asthma symptoms that children report every day. Because the graphs are too complicated for children aged <12 years to interpret on their own, they are shown only on the health care providers’ dashboard and not in children’s smartphone app.

##### Awareness of PA and Symptoms

Children are rewarded for the combination of filling out that they are going to be physically active and actually doing so. Moreover, the activities that are registered by the children become visible in an overview of performed activities in their app. Health care providers who tested a prototype indicated that children must learn to recognize their asthma symptoms and the relation among their asthma symptoms, PA, and medication. Therefore, the children’s asthma symptoms are monitored by reporting how they feel every morning and evening. On the health care provider’s dashboard, these scores are combined with the children’s PA behavior.

##### Suggestions for PA

In the needs assessment, children indicated that they often have motivation to become physically active, but they simply do not know what to do. Therefore, the app has a button that randomly shows suggestions from a list of PAs. The list contains both personalized and general ideas based on input of children participating in the needs assessment and testing the prototype. The personalized items can be added and adjusted every coaching moment with the health care provider.

##### Tips and Information

According to the needs assessment, health care providers and parents find it important that children learn more about the relation among their asthma symptoms, PA, and medication. Information regarding these topics are shown in informative and attractive drawings together with short supporting texts with practical tips for everyday life. These drawings pop up after children fill out that they are going to perform a PA in their app.

##### Schedule PA

A weekly schedule is visible in the app and the current day’s schedule is shown every morning.

##### Personal Coaching of Health Care Provider

Every week, children speak to their health care provider either in person or via a video call. On the basis of the visualizations on the health care providers’ dashboard, they discuss the child’s PA behavior of the past week, including the relation between the performed PA and the registered asthma symptoms. Moreover, the health care provider can adjust the personalized items in the dashboard such as the child’s PA goals, the informational topics, and the PA schedule.

### Step 5: Creating an Implementation Plan

#### Technical Structure

**Figure 2:**
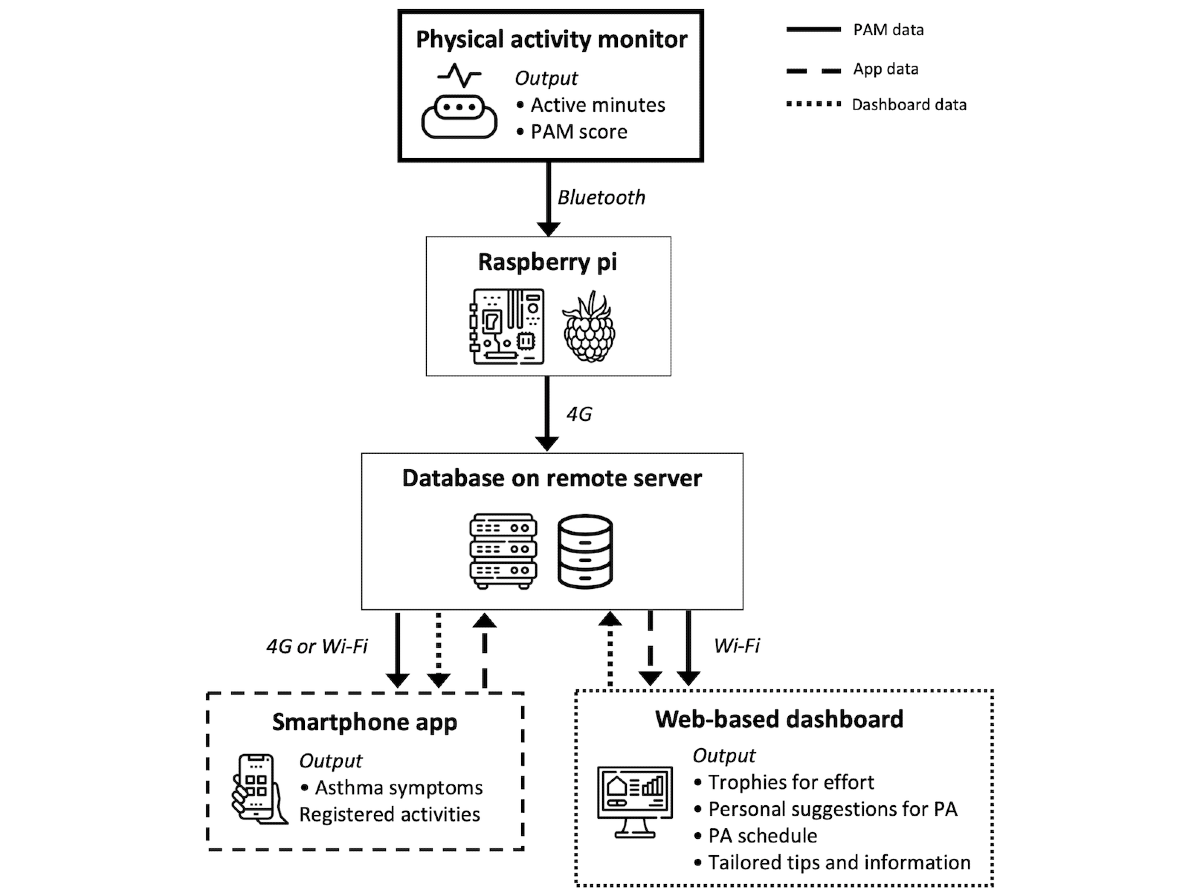
Overview of all technical elements. The arrows indicate data traffic between elements. PA: physical activity; PAM: physical activity monitor.

The three elements of *Foxfit* (the activity tracker, smartphone app for children, and web-based dashboard for health care providers) must communicate with each other to synchronize. Figure 2 shows an overview of the connections among all technical elements.

##### PA Tracker

Children wear an activity tracker that monitors their PA levels. After comparing several activity trackers, we chose the PA monitor (PAM) “PAM AM300” [79]. This instrument is shown to be valid for adults [80]. Although there is no information on the validity of the PAM for children, we expected the tracker to be applicable because in our intervention the PAM is primarily used to measure progress and relative differences. Moreover, the instrument met several important criteria for our intervention. The instrument gave the opportunity to send data to our private research database instead of a commercial organization, had positive reviews from young study participants, is reasonably priced, and is extremely easy to use, because it does not require charging and synchronizes automatically [81,82].

Children wear the PAM on their hip, where it is clipped on their waistband. The PAM measures their activity in 2 ways. First, their activity is represented as a PAM score indicating the ratio of energy expended through PA to resting metabolism [83]. Second, their daily activity is represented as active minutes per day. Those active minutes are classified based on the metabolic equivalent of task (MET) as either low intense (MET: 1.8-3), middle intense (MET: 3-7), or high intense (MET: >7). On the basis of this, bonus points are given: (1) 5 points for either 60 minutes of low intense activity or 90 minutes of middle activity (the health care provider chooses which rule fits the child best), (2) 5 points for at least 15 minutes of high intense activity, and (3) 5 points in case children registered an activity and their PAM tracks at least five middle or high intense activity minutes. Children’s total daily score is the sum of this PAM score and the gained bonus points.

To synchronize the smartphone app for children and the web-based dashboard for health care providers, the data collected from the PAM must be stored in a database. Therefore, children have a Raspberry Pi (Raspberry Pi Foundation) with a Bluetooth and 4G dongle at home. When the PAM is near the Raspberry Pi, it automatically sends the collected data to a database on a secured remote server via a secured Secure Shell (SSH) connection. The collected data are not shared with other parties, no personal data are stored, and the collected activity data are stored pseudonymized.

##### Web-Based Dashboard for Health Care Providers

**Figure 3:**
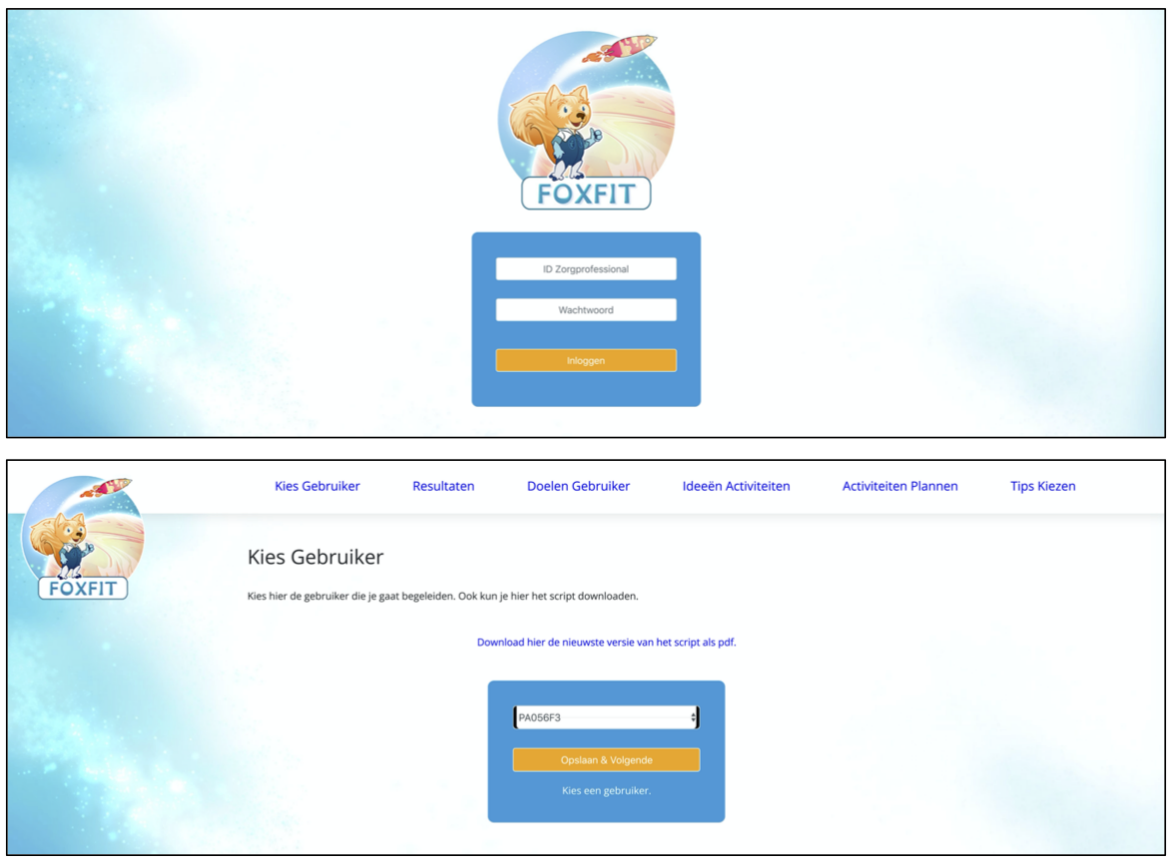
Start pages of the health care providers' web-based dashboard. Log in to dashboard (top); select the ID of the child (bottom).

Health care providers visit a web-based dashboard via their internet browser to see visualizations of the collected activity data and to personalize the intervention. Every health care provider has their own ID number and password to access the dashboard. These ID numbers and corresponding hashed passwords are stored in the database on the remote server. ID numbers of health care providers are only matched to ID numbers of children that they treat at the moment to ensure they do not have access to children who are treated by other health care providers. Figure 3 shows the pages of the dashboard where health care providers log in and select the child’s ID.

**Figure 4:**
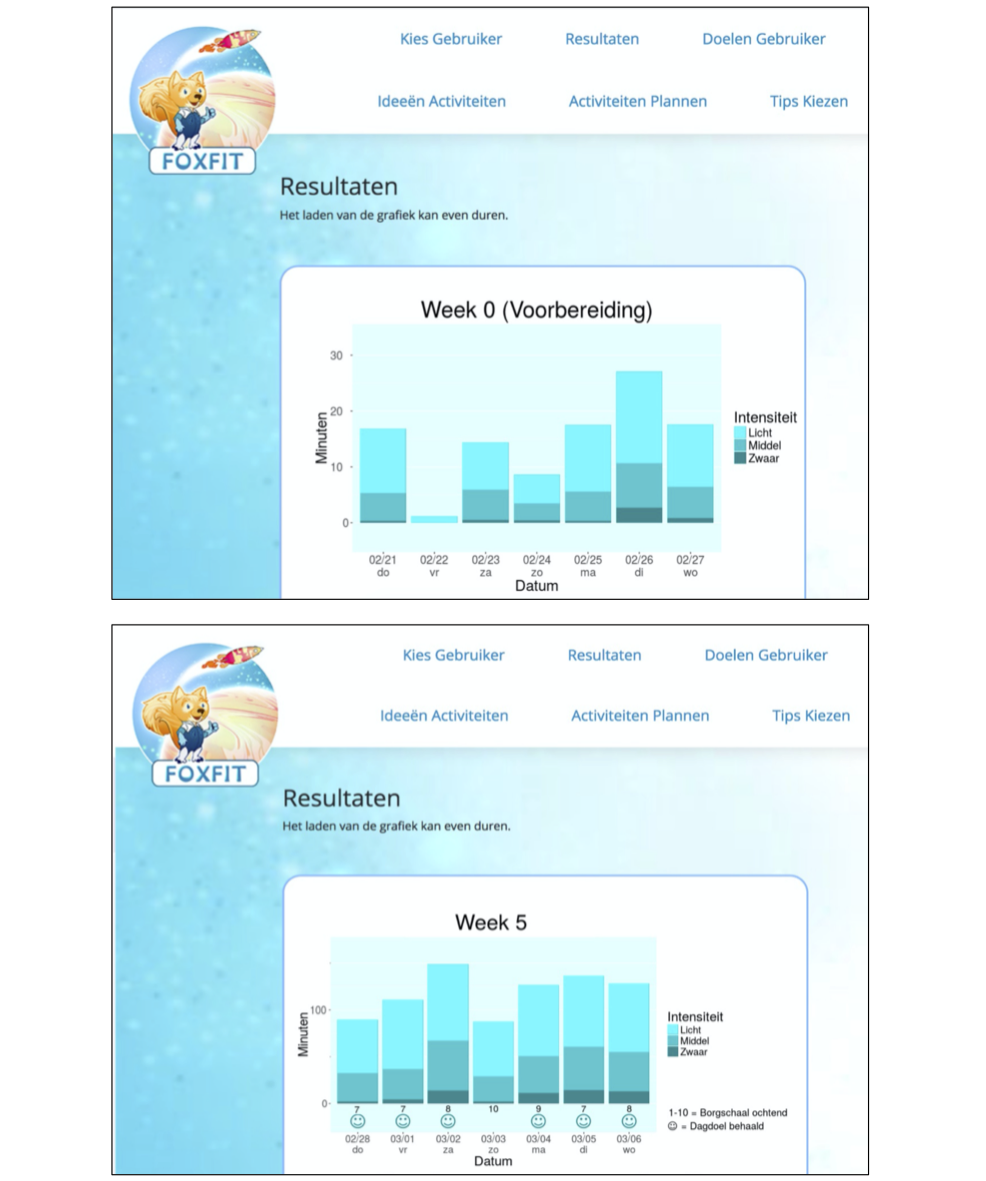
Visualization pages of the health care providers' web-based dashboard. Graphs regarding the physical activity (PA) behavior of the child are shown. From top to bottom: PA behavior of the baseline week (“Week 0”); PA behavior of the fifth week using the app (“Week 5”). In both graphs, the date is shown on the x-axis (“datum”), the y-axis represents the amount of PA minutes (“minuten”), and the intensity of PA (“intensiteit”) is represented by the color—low (“licht”); middle (“middel”); high (“zwaar”). In the bottom graph, the smiley faces indicate whether the child has achieved their daily goal, and the number between 1 and 10 indicates the experienced asthma symptoms in the morning (1=feeling very bad; 10=feeling very well).

**Figure 5:**
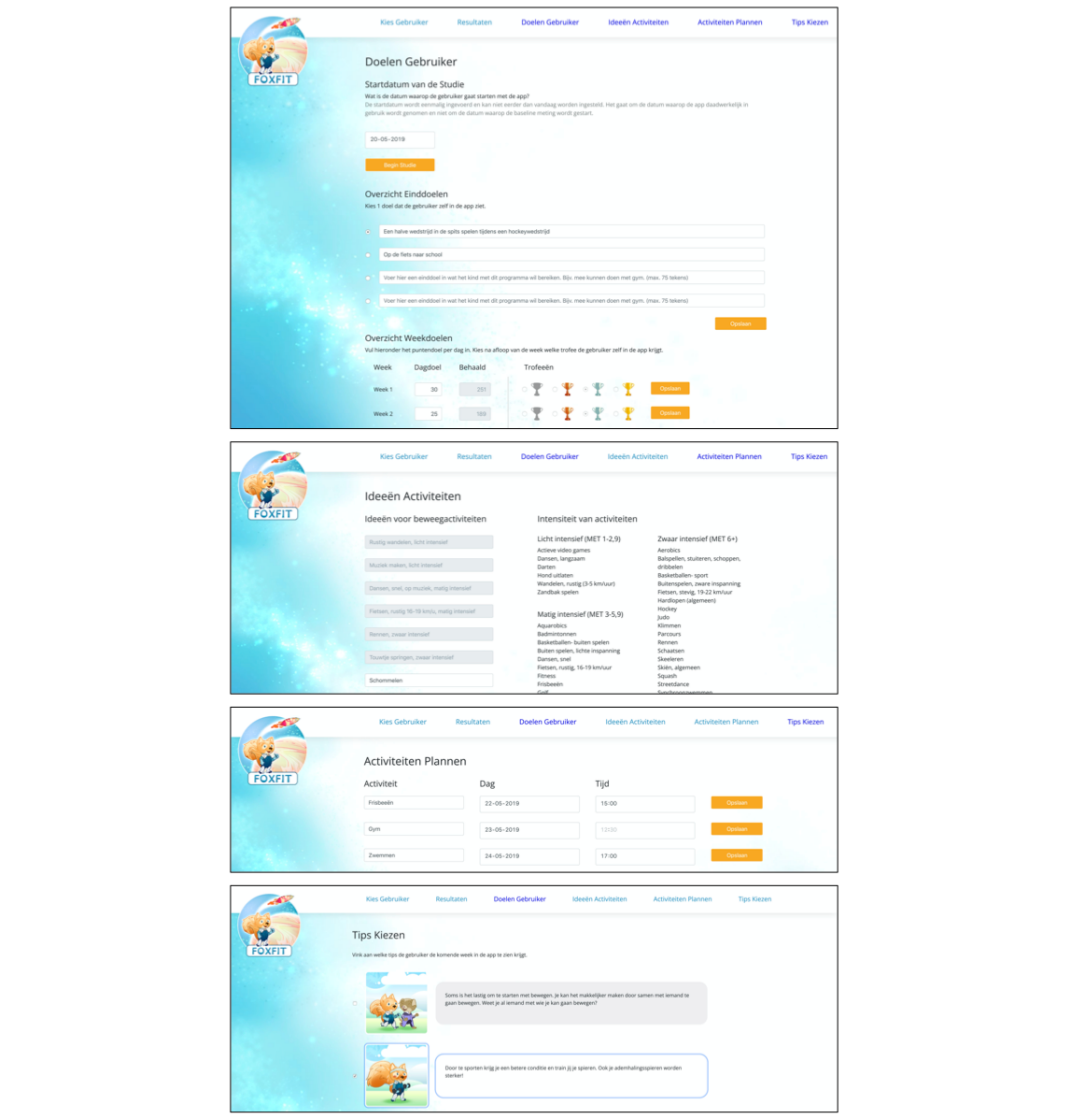
Personalization pages of the health care providers' web-based dashboard. From top to bottom: personal goals and trophies; physical activity (PA) suggestions; scheduling PAs; choosing relevant tips.

When a health care provider signs in to the dashboard and selects a child’s ID, the child’s activity data are sent via a secured SSH connection from the database on the remote server to the web-based dashboard. There, visualizations of the child’s PA behavior are shown. Figure 4 shows such visualizations on the health care provider’s dashboard. Health care providers also use the dashboard to tailor the intervention. Figure 5 shows pages of the dashboard in which the health care provider can fill out personalized data. When they add information or fill out choices, this is again sent via a secured SSH connection from the web-based dashboard to the database on the remote server.

##### Smartphone App for Children

**Figure 6:**
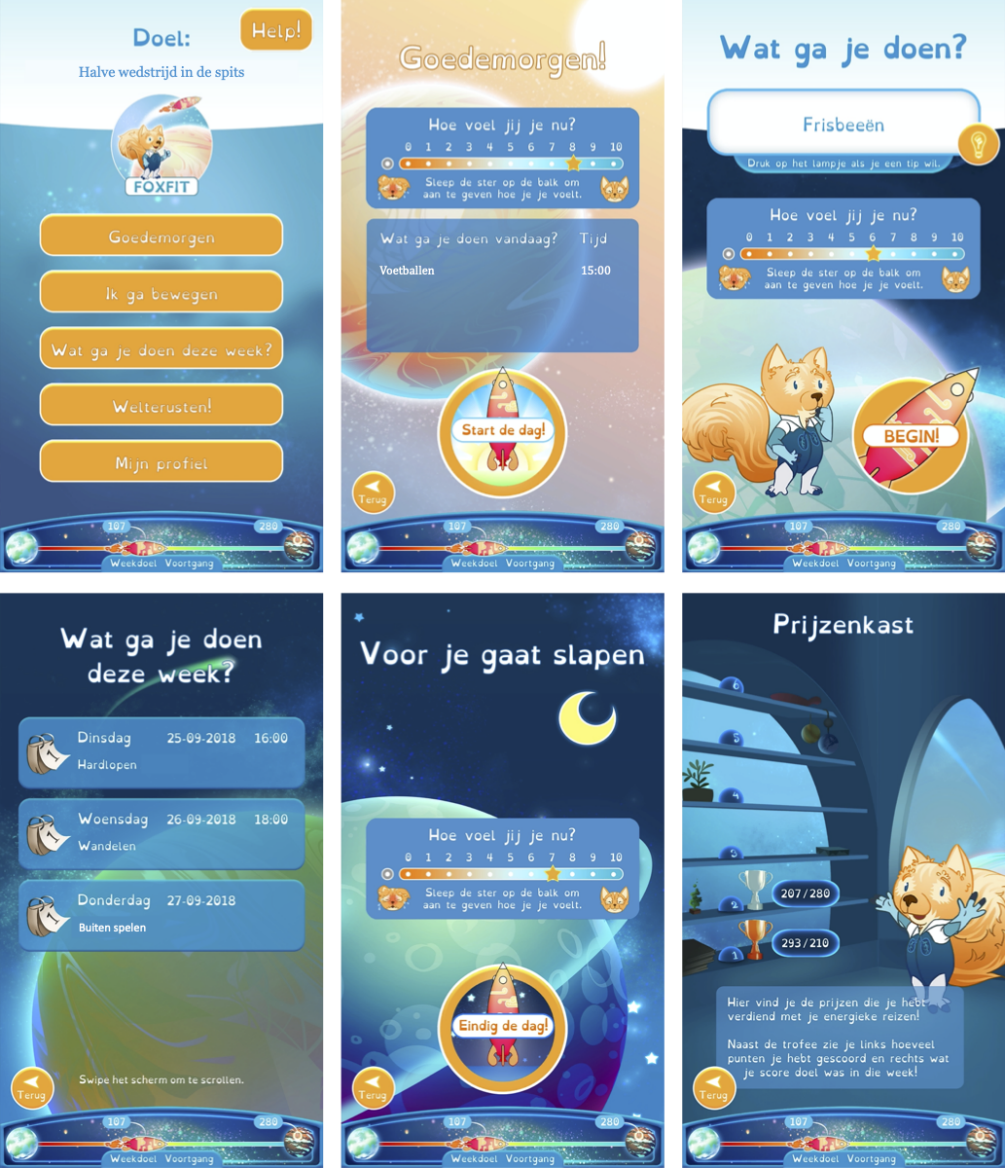
Pages of the smartphone app for the children. From left to right and from top to bottom: home screen; starting the day and filling out symptoms experienced in the morning; filling out physical activity (PA); overview of PAs of the current week; ending the day and filling out symptoms experienced during the day; earned trophies.

Because the *Foxfit* app is built only for Android, and because we wanted to prevent from privacy and installation problems, children receive a smartphone on which the *Foxfit* app is already installed. To see the collected activity data as a gamified story, children use the app. Figure 6 shows the pages of the child’s smartphone app. Children have their own ID number and password to access the app and the app is connected with the internet through Wi-Fi or 4G. On the remote server, no personal data of the child are stored. Only the ID number and corresponding hashed password are stored. When a child opens the app, activity data from the PAM that were automatically synchronized via the Raspberry Pi at home are sent from the remote server to the smartphone app through a secured SSH connection. When children add information or fill out choices in the app, this is also sent via a secured SSH connection from the smartphone app to the remote server. The home screen contains a help button that provides contact information to handle technical problems.

#### Implementation in the Health Care System

**Figure 7:**
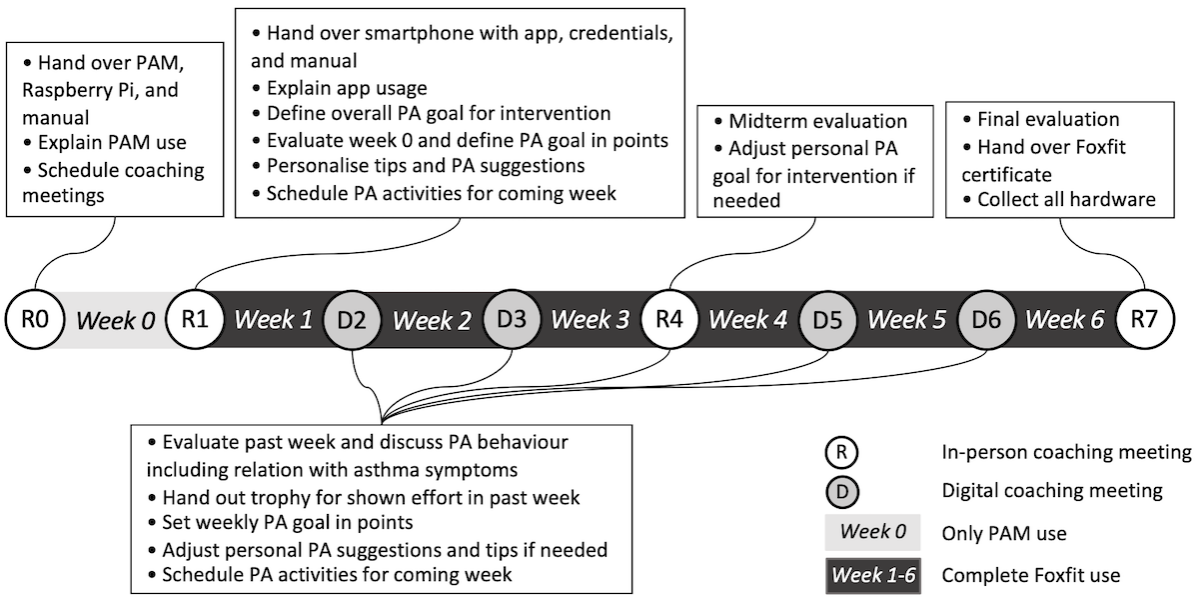
Timeline of the *Foxfit* intervention in the daily practice of the health care providers. PA: physical activity; PAM: physical activity monitor.

The intervention takes 7 weeks in total. Health care providers have a coaching moment with the child every week. The first 2 meetings, the midterm meeting, and the last meeting are in-person sessions and take 30 minutes. The remaining meetings are digital, to diminish the time investment and take 15 minutes. Before starting the intervention, children wear the PAM for a week without having access to the app. In this “week 0,” their baseline PA behavior is discovered. In the following 6 weeks, the full intervention including the smartphone app is used by the child. The health care provider can access the web-based dashboard at any time in the 7 weeks. At the start of a coaching meeting, the health care provider signs in into the web-based dashboard with their own credentials and selects the ID number of the child that the meeting is with. The dashboard is designed in such a way that the health care provider is guided through all steps to be taken. All steps and tasks to be performed by the health care provider are described in a manual as well. Figure 7 shows a timeline of the *Foxfit* intervention from the health care provider’s perspective.

In the first meeting, health care providers hand over the PAM, Raspberry Pi, and a manual to the child. They introduce the intervention and explain the PAM use to the child and their parents. Moreover, they schedule coaching moments for the coming weeks. After the baseline week is complete, health care providers hand over a smartphone on which the *Foxfit* app is installed, along with the child’s credentials and a manual. They explain the intervention to the child and their parents. Together with the child, a personal PA goal for the entire intervention is set as well as a weekly goal in the form of activity points. These goals are filled out in the dashboard. Moreover, personal tips and suggestions for PAs are chosen, and PAs for the coming week are scheduled.

In the digital coaching meetings, health care providers evaluate the PA behavior of the child over the past week, based on the PA graphs on their dashboard. They also discuss the relation between the child’s PA levels and their asthma symptoms. For the child’s shown effort in the past week, they hand out a trophy. Together with the child, the health care providers set a new weekly PA goal formulated as activity points and schedule PAs for the coming week. If needed, PA suggestions and personal tips can be adjusted in the dashboard as well.

The coaching meeting after 3 weeks of using the app is conducted in real life, instead of being conducted digitally. Then, health care providers conduct a midterm evaluation in addition to the general items covered during a coaching moment. If needed, they can adjust the child’s PA goal. In the last meeting, health care providers conduct a final evaluation in which they discuss the child’s progress achieved during the intervention. In addition, they hand out a *Foxfit* certificate to the child and they collect all hardware.

#### Implementation in Children’s Everyday Life

**Figure 8:**
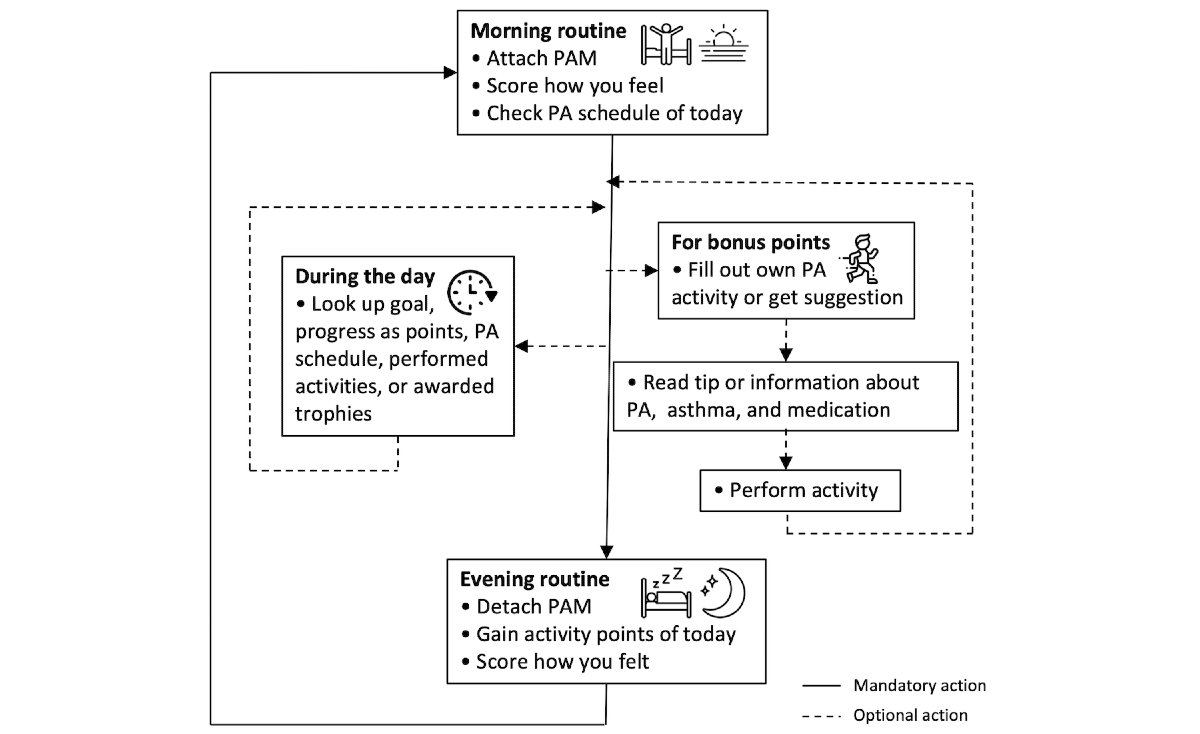
Timeline of the *Foxfit* intervention in the everyday life of the children. The arrows indicate flows between elements. PA: physical activity; PAM: physical activity monitor.

After the baseline week in which children’s baseline PA behavior is discovered with the PAM, children also use a smartphone with the *Foxfit* app. Children need not carry around their smartphone all the time. To minimize screen time, the app is not required to track PAs, because the PAM automatically tracks their PA levels. When they are near the Raspberry Pi at home, data are automatically sent to the database on the remote server. Figure 8 shows a timeline of the *Foxfit* intervention in children’s everyday life.

In the morning, children attach their PAM to their waistband to track their activity during the day. After that, they open the smartphone app which shows the menu and their personal PA goal. Then, they fill out how they feel that morning to track their asthma symptoms. When they are finished, their PA schedule of the day is shown, and they can start their day.

Although the app is not required to track PA behavior, children can use the app at any moment to register their PAs to become more aware of their own PA behavior. To do so, they open the app and fill out the activity they intend to perform at that moment. After registering, they receive a tip or information on subjects applicable to them. Children can use the app at any moment to look up their personal goal, received points, PA schedule, performed activities, or awarded trophies as well.

Around bedtime, children finish the *Foxfit* day by taking off the PAM and by gaining their daily points. When they open the app, they see the points received during the day. Their spaceship travels from the moon toward the planet based on the received PA points. Similar to the morning routine, children fill out how they felt during the day to track their asthma symptoms.

### Step 6: Creating an Evaluation Plan

The developed intervention *Foxfit* will be tested by approximately 15 children (aged 8 to 12 years) with asthma who are inactive. They will all be treated in a hospital in the Netherlands, specialized in pediatric asthma. During the evaluation study, the intervention will be provided by pediatric lung nurse practitioners and functional exercise therapists from those hospitals.

To evaluate the usability and feasibility in children’s everyday life and in health care providers’ daily practice, both children and health care providers will be interviewed halfway and at the end of their participation about their user experience, adherence, and suggestions for improvement. In addition to the qualitative interviews, they also fill in the System Usability Scale, which measures the usability of the developed intervention [84,85].

To obtain insights into the effect of the intervention, children will fill out several questionnaires at the start and end of the intervention. Those questionnaires regard asthma control, PA participation, enjoyment of PA, and self-perceptions with, respectively the Asthma Control Questionnaire, Activity Questionnaire for Adults and Adolescents, PA enjoyment scale, and the self-perception profile for children (competentie-belevingsschaal voor kinderen) [86-91]. Regarding children’s physical fitness and endurance, they perform a 6-minute walk test. In addition, activity data, collected with the PAM, of all participants will be analyzed. We will compare activity levels of the baseline week with activity levels of the last intervention week.

A detailed description of the evaluation together with its corresponding analyses and results will be published separately.

## Discussion

### Principal Findings

This study illustrated the way IM was used to translate theory and stakeholder experiences into design requirements and functional components and finally develop a complete blended and technology-supported intervention. IM was useful for describing the translation and development in a structured way. The needs and wishes of children with asthma, their parents, and health care providers were combined with scientific evidence regarding behavior change techniques and gamification in a technology-supported intervention. This resulted in the blended intervention *Foxfit* that consists of an activity tracker, a smartphone app for children (aged 8 to 12 years), and a web-based dashboard for health care providers. It focuses on PA in everyday life to improve social participation and contains behavior change elements such as goal setting, rewards, action planning, monitoring, shaping knowledge, a tailored approach, personal coaching and feedback, and a gamified story. Because of the cocreation with future users, the support of the resulting product has been strengthened among children with asthma, their parents, and health care providers. The structured description of the development process ensures that *Foxfit* can be compared easily with other interventions.

### Strengths, Limitations, and Future Work

The participation of stakeholders in this project was very strong, because the intervention was not only created for the target group but together with them. Consistent with other studies that applied IM to develop health interventions for children, we experienced that the process of IM is very time consuming, but the systematic approach improves clarity and analysis of the intervention [41,42]. The theoretical foundation increases the potential of the intervention to realize the desired outcome of supporting PA, because it tends to provoke more significant effects on PA [30,65,92,93]. Moreover, clarity and analysis of the intervention is important, because many gamified interventions for children with chronic disease lack a detailed description of the final intervention [61].

Because of the structured approach, it is relatively easy to apply this approach to other situations. On the one hand, the current gamified story and integrated behavior change techniques can be applied for children with other chronic diseases. Only the content of the knowledge components and the specific questions about asthma symptoms should be adjusted to the new disease. On the other hand, the integrated behavior change techniques can be applied for children in older age groups in case the gamified story is adjusted to their interests and experiences. Because children should be able to handle a smartphone with the *Foxfit* app themselves and understand why they need to wear the activity tracker, we do not think that the intervention is suitable for children younger than the current minimum age of 8 years.

An important factor of *Foxfit* is the support of the health care provider. Because of the blended approach, children receive personal feedback, and the intervention can be tailored. These components are success factors when behavior change is aimed for and requires considerable time from both children and health care providers [67-69]. The intervention takes 7 weeks, which will not be long enough to reach lasting behavior change. However, 7 weeks of monitoring and coaching can lead to positive PA experiences and therefore, improved PA levels. Extended use of the intervention, possibly with decreasing involvement of the health care provider, can help maintain these improved PA levels and form new habits. In the evaluation study we will obtain an indication of the effect of the intervention on improved PA levels.

Time is scarce and expensive for the health care providers. Therefore, we tried to diminish the coaching time by enabling web-based coaching meetings and by minimalizing health care providers’ preparation time. Despite these optimizations, it might be difficult to structurally implement the *Foxfit* intervention in the health care system. Because of the advantages of personal feedback from health care providers on children’s motivation, we would not recommend to completely omit this. However, the investment time of health care providers might be further diminished through the use of artificial intelligence (AI) for automatically personalizing the intervention. Such a technical system might, for instance, suggest health care providers weekly activity goals based on activity data of the past weeks. This approach takes the attitude of both patients and health care providers toward AI into account. Patients appreciate the human factor of health care providers and might have a negative attitude toward medical AI because of the absence of this humanistic care factor [94]. Moreover, health care providers appreciate AI systems to make the process more efficient or support them by giving suggestions, but ultimately, they want to make their own choices [95]. By giving AI-based suggestions for weekly PA goals, there still is clinical expertise and personal feedback, but the health care providers are supported in their decisions and the process is made more efficient.

Although coaching from the health care provider is an important component of the intervention, it would be interesting to study whether the intervention can be used as standalone app for children as well. Although the intervention was designed for children with asthma who are inactive, there are also several children who already have moderate PA levels but who may still be able to diminish the impact of their disease when their PA levels are improved. A stand-alone app might, for instance, be helpful for them as well as for children who completed the entire blended *Foxfit* intervention previously and now need a repetition. To examine the possibilities of a standalone app, we should study whether some of the health care provider’s tasks and dashboard functionalities can be implemented in the smartphone app. Moreover, in some cases, highly educated parents could possibly take over the coaching role of the health care provider when they receive proper instruction.

Regarding the hardware, the PAM that children must wear is extremely easy to use and safe in terms of personal data. No personal data are stored, and the activity data are not shared with a commercial organization. The PAM does not require charging during the intervention and it synchronizes automatically. Thus, the impact on children’s everyday life is minimal. However, this system requires Raspberry Pi with Bluetooth and 4G dongle at home. This takes up space, requires an electrical outlet, and comprises of small components, which might be inconvenient when having very young children at home.

Although a large group of children participated in the needs assessment, it was challenging to include children with low PA levels for our pilot test. In the end, more children with sufficient PA levels than children with low PA levels pilot-tested our prototype than children with low PA levels. This might have influenced the feedback of the prototype and therefore the choices made for the final intervention. For example, in the needs assessment, children suggested that competitive elements would help them become physically more active, whereas participants of the stakeholder sessions indicated that competition is not desired, because children’s motivation might be reduced when they compete against children having better PA possibilities. A solution might be to compete against each other based on children’s personalized goals. In the feasibility and usability tests, user experiences and suggestions for improvement of a larger group of children with asthma who are inactive will be explored.

According to the Fogg Behavior Model, someone will not automatically perform the desired behavior when the opportunity, capability, and motivation are sufficient, but there will be a moment of opportunity in which someone can be persuaded to perform the desired behavior in case he receives a trigger [96,97]. In technology-supported interventions, providing such an external trigger at the moment of opportunity is called a just-in-time notification. Although such notifications might have reinforced children’s motivation, we did not choose to implement them, because one of our requirements was that the intervention should be usable without the children having to carry the smartphone with them all day. The PAM did not technically support such notifications, but incorporating these just-in-time notifications might strengthen the motivational aspect of our intervention in the future.

### Conclusions

IM was useful to structure the development of the blended and technology-supported intervention *Foxfit*, and to describe this development process. *Foxfit* facilitates health care providers in promoting PA in children with asthma. The intervention consists of an activity tracker, smartphone app for children, and web-based dashboard for health care providers. Important behavior change elements of the intervention are goal setting, rewards, action planning, monitoring, shaping knowledge, a gamified story, personal coaching and feedback, and a tailored approach. Not only is the intervention based on behavior change techniques, it also meets the needs and wishes of its users because it was cocreated with children with asthma, their parents, and their health care providers.

## Abbreviations

AI: artificial intelligence
CeHReS: Centre for eHealth Research
IDEAS: Integrate, Design, Assess, and Share
IM: intervention mapping
MET: metabolic equivalent of task
MRC: Medical Research Council
PA: physical activity
PAM: physical activity monitor
SSH: Secure Shell
